# Identification of Genes and Pathways Related to Phenol Degradation in Metagenomic Libraries from Petroleum Refinery Wastewater

**DOI:** 10.1371/journal.pone.0061811

**Published:** 2013-04-18

**Authors:** Cynthia C. Silva, Helen Hayden, Tim Sawbridge, Pauline Mele, Sérgio O. De Paula, Lívia C. F. Silva, Pedro M. P. Vidigal, Renato Vicentini, Maíra P. Sousa, Ana Paula R. Torres, Vânia M. J. Santiago, Valéria M. Oliveira

**Affiliations:** 1 Microbial Resources Division, Research Center for Chemistry, Biology and Agriculture, Campinas University, Campinas, Sao Paulo, Brazil; 2 Department of Primary Industries, BioSciences Research Division, Melbourne, Victoria, Australia; 3 Laboratory of Molecular Immunovirology, Department of General Biology, Federal University of Viçosa, Viçosa, Minas Gerais, Brazil; 4 Bioinformatics Laboratory, Institute of Applied Biotechnology to Agriculture, Federal University of Viçosa, Viçosa, Minas Gerais, Brazil; 5 Genetic Engineering and Molecular Biology Center (CBMEG), Campinas University, Campinas, Sao Paulo, Brazil; 6 PETROBRAS R&D Center, CENPES, Rio de Janeiro, Rio de Janeiro, Brazil; Université Paris-Sud, France

## Abstract

Two fosmid libraries, totaling 13,200 clones, were obtained from bioreactor sludge of petroleum refinery wastewater treatment system. The library screening based on PCR and biological activity assays revealed more than 400 positive clones for phenol degradation. From these, 100 clones were randomly selected for pyrosequencing in order to evaluate the genetic potential of the microorganisms present in wastewater treatment plant for biodegradation, focusing mainly on novel genes and pathways of phenol and aromatic compound degradation. The sequence analysis of selected clones yielded 129,635 reads at an estimated 17-fold coverage. The phylogenetic analysis showed Burkholderiales and Rhodocyclales as the most abundant orders among the selected fosmid clones. The MG-RAST analysis revealed a broad metabolic profile with important functions for wastewater treatment, including metabolism of aromatic compounds, nitrogen, sulphur and phosphorus. The predicted 2,276 proteins included phenol hydroxylases and cathecol 2,3- dioxygenases, involved in the catabolism of aromatic compounds, such as phenol, byphenol, benzoate and phenylpropanoid. The sequencing of one fosmid insert of 33 kb unraveled the gene that permitted the host, *Escherichia coli* EPI300, to grow in the presence of aromatic compounds. Additionally, the comparison of the whole fosmid sequence against bacterial genomes deposited in GenBank showed that about 90% of sequence showed no identity to known sequences of Proteobacteria deposited in the NCBI database. This study surveyed the functional potential of fosmid clones for aromatic compound degradation and contributed to our knowledge of the biodegradative capacity and pathways of microbial assemblages present in refinery wastewater treatment system.

## Introduction

Phenol and phenolic compounds are the main organic pollutants discharged into petroleum refinery wastewater. Besides causing serious damage to the environment and living beings, low concentration of these compounds can inhibit the growth of microorganisms present in biological wastewater treatment systems, consequently promoting a significant reduction in the rate of degradation of contaminant compounds [1], [2]. Therefore, there is a need for petroleum refineries to remove phenol from their effluents [1].

Several processes have been used for the elimination of phenolic compounds, with biological treatments being preferred to physicochemical treatments. Biological treatments have been successful because they are a green process, which use microorganisms found in the environment to mineralize pollutant compounds in water and CO_2_, in addition to their low operational costs [3]. As a result, several microorganisms which degrade phenolic compounds have been studied, such as the bacterial genera *Acinetobacter*, *Alcaligenes*, *Thauera*, *Azoarcus*, *Comamonas*, *Pseudomonas*, *Bacillus* and some yeasts, such as *Candida tropicalis* [2], [4]–[7]. These bacteria use a typical aerobic degradation pathway of phenolic compounds, which has two critical steps: i) the ring hydroxylation of adjacent carbon atoms and ii) the ring cleavage of the resulting catecholic intermediates. In the phenol degradation, particularly, the aromatic ring is initially monohydroxilated in the adjacent carbon of a hydroxyl group by the enzyme phenol hydroxylase (PH, phenol 2- monooxygenase, EC 1.14.13.7) resulting in catechol, which is in turn cleaved by either *ortho*- or *meta*-cleavage pathway. In case of the *ortho*-pathway, the ring is cleaved by the catechol 1,2-dioxygenase enzyme (C12O), leading to the initial formation of succinyl-CoA and acetyl-CoA. In the *meta*-pathway, the catechol is cleaved by the catechol 2,3-dioxygenase enzyme (C23O), leading to the formation of pyruvate and acetaldehyde (Marimaa *et al.*, 2006). There are two types of bacterial phenol hydroxylases, the simple (sPH) and the multicomponent (mPHs) enzymes, with the latter being the most frequently found in the environment [8].

Several studies of phenol microbial catabolism have been conducted to better understand the degradation of aromatic compounds [9]. However, a few studies were done using the metagenomic approach, which involves the construction of fosmid libraries containing large DNA fragments from the environmental microbial community. The resulting libraries can be screened for several interesting target sequences and functions in the search for new products, genes or metabolic pathways from mainly uncultivated microorganisms [10], [11]. One of the most complete studies on aromatic compound degradation pathways of uncultivated bacteria was carried out by Suenaga *et al.* (2009), through the construction of a metagenomic library from activated sludge of treat coke plant wastewater. The authors analyzed 38 fosmid clones and found 36 clones carrying novel gene arrangements of extradiol dioxygenase, a key enzyme in the degradation of aromatic compounds. The present study aimed to identify genes and metabolic pathways related to degradation of phenol and other aromatic compounds in sludge samples from a petroleum refinery wastewater treatment system, using a metagenomic approach to capture a broader range of the extant functional diversity.

## Materials and Methods

### Sampling and phenol acclimation

Sludge samples were collected from two different refinery wastewater treatment plants of the petroleum industry Petrobras (Brazil). One of the samples, MBR1, was collected from a laboratory-scale (2 L) continuous membrane bioreactor (MBR) after a 30-day period of high phenolic load feeding (68.5 mg L^−1^), as previously described by Viero and collaborators [12]. The second sludge sample, MBR2, was collected from a pilot submerged membrane bioreactor, previously described by [13], and subjected to acclimation in batch culture for a 30 day-period up to 1.0 g L^−1^ of phenol (Merck, USA). The acclimation step was performed in triplicate using 2.0 g L^−1^ of sludge as inoculum added to an Erlenmeyer flask containing 300 mL of an initial rich nutrient medium (2.75 g L^−1^ K_2_HPO_4_, 2.25 g L^−1^ KH_2_PO_4_, 0.1 g L^−1^ NaCl, 1.0 g L^−1^ (NH_4_)_2_SO_4_, 0.2 g L^−1^ MgCl_2_.6H_2_O, 0.01 g L^−1^ CaCl_2_ and 1 g L^−1^ yeast extract as carbon source). These flasks were incubated at ambient temperature and on an orbital shaker (150 rpm) to provide aerobic conditions. The initial carbon source was gradually diminished and replaced with phenol, in the proportion of 0.5 g L^−1^ decrease of yeast extract for each 0.2 g L^−1^ increment of phenol, until the yeast extract was totally eliminated. The sludge was collected after the microorganisms were considered totally adapted to 1.0 g L^−1^ phenol, *e.g.* when 100% phenol was removed in less than 24 hours. The phenol was chosen as an aromatic model compound and used as a sole carbon source to evaluate the growth of *Escherichia coli* fosmid clones. The acclimated batch culture was done as an enrichment strategy, in order to increase the probability of identifying metagenomic clones containing genes related to phenol degradation.

### Nucleic acid extraction and metagenomic fosmid library construction

High molecular weight DNA extraction from sludge samples was carried out using the protocol previously described by Silva *et al.* [14]. The two metagenomic libraries, one for each sludge sample, were constructed using the CopyControl™ HTP Fosmid Library Production Kit (Epicentre, USA), according to the manufacturer's instructions. First, the metagenomic DNA was separated using pulsed field gel electrophoresis and 25–50 kb DNA fragments were excised, purified, blunt-ended and ligated into the pCC2FOS fosmid vector contained in the kit. The ligation reaction was then packaged into lambda phage using MaxPlax Lambda Packaging Extracts, and then the packaged library was transformed into *Escherichia coli* EPI-300 T1^R^. Transformants obtained were selected on LB agar plates containing 12.5 µg mL^−1^ chloramphenicol (LB/Cm). The clones were transferred to 96-well microtiter plates containing LB/Cm and glycerol 20%, and stored at −80°C.

The validation of the metagenomic libraries was carried out using six fosmid clones randomly selected from each library. The fosmid DNA from each clone was extracted using the FosmidMax DNA Purification kit (Epicentre, USA), according to the manufacturer's protocol, and then digested using 10 U *NotI* restriction enzyme (Promega, USA) at 37°C overnight. The band profiles of fosmid clones were checked in preparative pulsed field gel electrophoresis (*Pulsed-field CHEF DRIII System -* BioRad- USA) at angle 120°, 6 Vcm^−1^, 1 s–12 s switch time, 10.5 h at 14°C.

### Screening the metagenomic library for phenol hydroxylase gene

Clones from both metagenomic libraries were cultured in 96-well microplates containing 150 µL LB/Cm broth and incubated at 37°C on a rotary shaker (150 rpm) for 16 h. Each twelve clone cultures were pooled and used as template for the PCR screening. These master pools were obtained by adding 2 µL of each clone culture in 10 µL of MILLI-Q water, and 5 µL-aliquots of the master pool was used for PCR amplification. The PCR was done in 96-well microplates containing 0.5 pmol/µL each primer pheUf and pheUr, used for the amplification of the largest subunit of the multicomponent phenol hydroxylase (LmPH) gene [15], 0.2 mM dNTPs, 1× Tris-HCl, 0.1 mM BSA and 2.5 U *Taq* DNA Polimerase (Invitrogen, USA) in a final volume of 50 µL. The amplification conditions were 10 min at 94°C, 5 cycles consisting of 1 min at 94°C, 1 min at 58°C and 1 min at 72°C, followed by 25 cycles of 1 min at 94°C, 1 min at 56°C and 1 min at 72°C; and a final extension of 10 min at 72°C. A second PCR screening, performed using the same reaction and amplification program conditions described above, was carried out with individual clones from each of the positive pools in order to identify the positive clone.

### Screening the metagenomic library for phenol degradation activity

The functional screening assays were developed according to the methodology described by Johnsen *et al.* [16] with some modifications. Clones from both libraries were pre-cultured in 96-well microplates containing 150 µL LB broth added of chloramphenicol (12.5 µg/mL) and incubated at 37°C on a rotary shaker (150 rpm) for 16 h. After growth, aliquots (15 µL) of the clone cultures were transferred to another microplate containing 150 µL Bushnell Haas (BH) mineral medium [17], [18] containing chloramphenicol (12.5 µg/mL) and phenol 0.02%, which was pre-sterilized by filtration through a 0.22 µM Millipore membrane. *Escherichia coli* EPI 300 cells were used as a negative control. After 48 h incubation at 37°C on a rotary shaker (150 rpm), 30 µL MTT [3-(4,5-dimethyl-2-thiazolyl)-2,5 -diphenyl-2 H-tetrazolium bromide] (Merck) solution (1 mg/mL) was added to each well to evaluate microbial respiration and consequent phenol consumption. The microplates were incubated again at 37°C for 1 h. The generation of a purple color was considered to be a positive hit, while a yellow color was indicative of the absence of cellular activity [19].

### Extraction of fosmid DNA pools and pyrosequencing

One hundred positive clones were randomly selected based on identification by sequence- and/or function-based screens for pyrosequencing analysis. The clone number was determined based on the coverage calculations of the pyrosequencing analysis. For fosmid extraction, these clones were grown in plastic tubes containing 5 mL of LB/Cm medium for 17 h at 37°C on an orbital shaker (180 rpm). After growth, these clones were pooled into a final volume of approximately 500 mL, which was used for fosmid extraction with the Large-Construction Kit (Qiagen, USA), according to the manufacturer's protocol. Finally, five µg of pooled fosmid DNA were used for the pyrosequencing technique, which was done according to the 454/Roche GS-FLX instruction protocols. The pyrosequencing step was done in collaboration with the Department of Primary Industries, BioSciences Research Division (Victoria/Australia).

### Assembly and sequence analysis of fosmid DNA pools

The sorting and trimming of the metagenomic data, based on the quality and size of the reads, as well as the contig assembling were done using the 454 Newbler assembler (version 2.0.01.14) for Genome Sequencer FLX (Roche, USA). All contig sequences ≥1,000 bp were selected and analyzed by the PRODIGAL program (Prokaryotic Dynamic Programming Genefinding Algorithm, http://prodigal.ornl.gov), which predicts all possible open reading frames (ORFs). These possible ORFs were annotated by using the MG-RAST and BLASTp platforms.

The metabolic pathway classification of all contig sequences was done using the MG-RAST platform (http://metagenomics.anl.gov). The data were submitted as text file (.txt) for online annotation using the subsystems technology. In this approach, reads are classified in a hierarchical structure in which all genes required for a specific function are grouped into subsystems [20].

The possible ORFs annotated as functions related to phenol degradation by MG-RAST were re-analyzed by BLASTp, followed by phylogenetic analyses using the softwares ClustalX [21] and MEGA (v. 4.0) [22]. After the sequence alignment, the evolutionary distance was calculated by MEGA, using the DNA substitution model described by Kimura [23]. The phylogenetic reconstruction was done using the neighbor-joining algorithm with bootstrap values calculated from 1,000 replicate runs, using the routines included in the MEGA software.

### Sequencing, assembly and sequence analysis of fosmid insert

One out of 100 positive clones was randomly selected for subsequent sequencing of the whole fosmid insert. This clone was chosen due to its positive response in the functional screening, thus there would be a higher probability to find new genes or pathways related to phenol or aromatic compound degradation. The selected clone was sent for sequencing by the company Macrogen (Seoul, Republic of Korea), using the shotgun library sequencing approach.

The assembly of the contig and the sequence analysis of the fosmid insert was performed using CLC Main Workbench Version 6.8.1 (CLC Bio). Sequences of the reads were trimmed by quality (Phred score >20), vector sequences were excluded, and one contig of 33.452 kb was assembled with 8.4 fold coverage. The possible ORFs were predicted using the Bacterial Genetic Code (NCBI translation table 11) and alternative start codons (AUG, CUG, and UUG), using CLC Main Workbench and PRODIGAL (http://prodigal.ornl.gov). All predicted ORFs were annotated using Blastx searches against GenBank database (http://www.ncbi.nlm.nih.gov/genbank).

### Prediction of protein-protein interactions

The dataset used to predict protein-protein interaction of contig 20 was composed of reads annotated as phenol hydroxylase subunits and catechol 2,3-dioxygenase, that showed at least 60% of coverage compared to the SwissProt protein sequences. The STRING database [24] was used to predict any putative protein interaction networks, providing an overview of the physical and functional associations and interactions between the proteins.

### Nucleotide sequence accession number

The raw data set from the fosmid libraries are available from the NCBI Short Read Archive (SRA047580.2). The fosmid insert sequence has been deposited in GenBank under the accession number KC747109.

## Results and Discussion

### Sequence and functional screening of fosmid metagenomic libraries

A total of 13,200 clones were obtained from both metagenomic fosmid libraries from wastewater treatment sludges, with 10,000 from the laboratory-scale membrane bioreactor and 3,200 from the acclimated sludge in batch culture. All clones were used for sequence- and function- based screening for phenol degradation. The PCR assays yielded 26 positive hits, each of which revealed an amplicon for the largest subunit of the phenol hydroxylase gene. From these, 12 clones were from the laboratory-scale membrane bioreactor and 14 were from the acclimated sludge batch culture.

The functional assay allowed the detection of 413 clones able to grow in mineral medium with phenol as a sole carbon source, with 211 clones from the laboratory-scale membrane bioreactor and 202 clones from the acclimated batch culture.

### Phylogenetic and functional profile of fosmid clones related to phenol degradation

In order to have high coverage of sequencing data, one-hundred positive clones were randomly selected and submitted to pyrosequencing. This analysis resulted in 129,635 sequences, approximately 63 Mbp of DNA, with average size of read lengths about 500 bp and GC percent about 60±8%. The majority of sequences showed the G+C content DNA percentage of 65% to 70%, this result showed that *E. coli* host strain, with an average G+C content DNA of 51%, offer no serious bias against pyrosequencing data analysis. Ninety-five percent of these reads formed contigs, totalizing 609 contigs. From these, 516 were greater than 500 bp and 341 were greater than 1,000 bp. These results showed that the pyrosequencing had 17-fold coverage, suggesting good perspectives of finding operons related with degradation of phenolic compounds.

**Figure 1:**
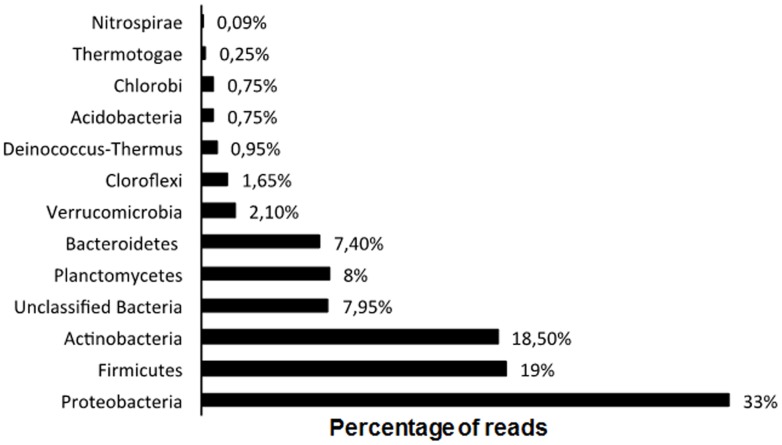
Phylogenetic classification based on metagenomic pyrosequencing data from phenol degrading clones by MG-RAST platform. (N = 129,635 reads).

All sequences were phylogenetically analyzed by the MG-RAST platform and results revealed that the majority of the sequences were affiliated to the Proteobacteria phylum, followed by Actinobacteria and Firmicutes phyla (Figure 1). Within the Proteobacteria phylum, the most abundant class was Betaprotebacteria, encompassing mainly the orders Burkholderiales (88.68%) and Rhodocyclales (6%). These results corroborate with literature data in the sense that the majority of the taxa related to phenol degradation are affiliated to the Proteobacteria phylum [4]–[7]. Nonetheless, these results prove once more the predominance of proteobacteria species playing important roles in the degradation of pollutant compounds. Additionally, the data obtained in the present work, derived from the pyrosequencing approach, are in accordance with a previous work published by our research group [13] based on the use of the 16S rRNA gene library methodology in both DNA and RNA MBR samples, that showed Burkholderiales and Rhodocyclales as the most abundant orders in the sludge as well.

**Figure 2:**
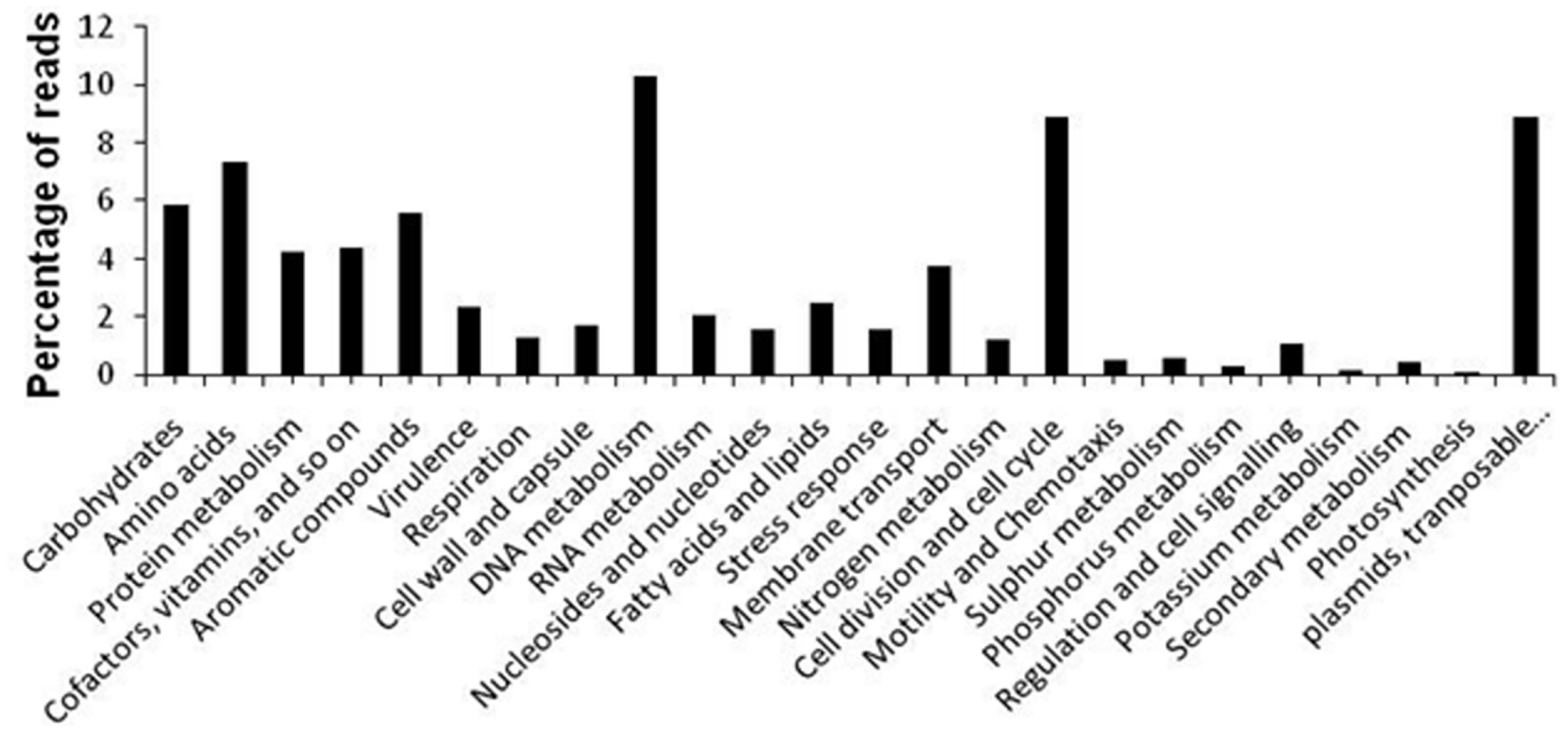
Metabolic profile based on metagenomic pyrosequencing data from phenol degrading clones by MG-RAST platform. (N = 129,635 reads).

The sequences derived from the pyrosequencing were also used to generate a metabolic profile of the clones with the MG-RAST plataform [25]. Using the BLASTx and E*-value* cutoff of 0.001, the metabolic profile showed a broad functional diversity, encompassing many functions required in wastewater treatments, such as the metabolism of phosphorous (0.32%), sulfur (0.66%), nitrogen (1.26%) and aromatic compounds (5.59%), in addition to housekeeping functions (Figure 2).

**Figure 3:**
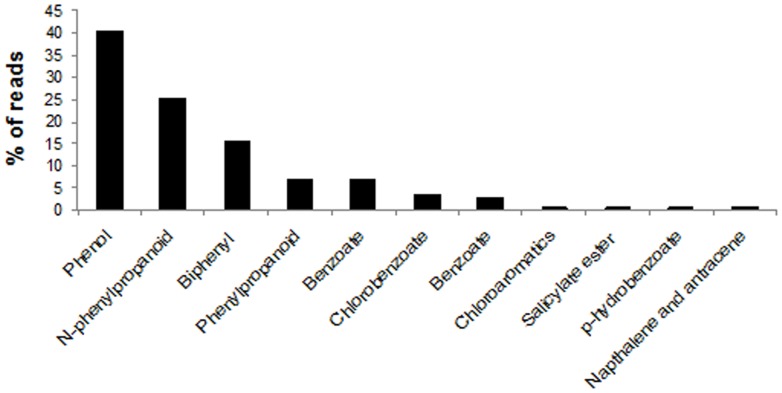
Profile of peripheral degradation pathways of aromatic compounds of metagenomic pyrosequencing data from phenol degrading clones by MG-RAST platform. (N = 129,635 reads).

The metabolism of the aromatic compound category was subdivided into several subcategories, with the majority of the genes found grouped into peripheral pathways for the catabolism of aromatic compounds (50.76%), followed by the metabolism of central aromatic intermediates (24.32%), anaerobic degradation of aromatic compounds (6.19%) and others. Important genes related to the degradation of phenolic compounds, such as catechol dioxygenase and phenol hydroxylase, were grouped into the central aromatic intermediates and peripheral pathways for the catabolism of aromatic compounds, respectively. In this last category, besides phenol hydroxylase, several genes related to important functions in pollutant degradation were also included, such as biphenyl degradation, benzoate catabolism, and naphthalene and anthracene degradation (Figure 3). Additionally, the functional profile revealed the presence of bacteria in the sludge with potential role for aerobic and anaerobic degradation of phenol by *meta*-cleavage, as well as for the degradation of many other aromatic compounds such as biphenyl, benzoate and naphthalene.

### Contig analysis of fosmid clones related to phenol degradation

Around three hundred and fifty non-redundant contigs larger than 1,000 bp were analyzed by using the PRODIGAL software with the purpose to search for all possible open reading frames (ORFs) from the dataset. The PRODIGAL analysis generated 2,276 possible ORFs, the majority of them with more than 700 bp in length. All open reading frames identified were translated into amino acid sequence and assigned a function based on homology by MG-RAST. This program assigned 63% of these ORFs, which were grouped into the same categories described in Figure 2. About 7.1% were grouped into the category of metabolism of aromatic compounds, which encompassed 25 ORFs affiliated to proteins related to phenol degradation, such as multimeric phenol hydroxylase and catechol dioxygenase.

These ORFs were selected for manually annotation by BLASTp followed by phylogenetic analysis. The most similar protein, identity and bacterial affiliation corresponding to these sequences are described in Table 1. These results showed that the pyrosequencing of clones was represented by four of the seven subunits of phenol hydroxylase multimeric enzyme: positive regulatory oxygenase component P1 (DmpL), P2 oxygenase component (DmpM), P3 oxygenase component (DmpN) and oxyreductase FAD-binding (DmpD). The catechol 2,3-dioxygenase enzyme belongs to the same pathway of the phenol hydroxylase multimeric enzyme, the *meta*-pathway, responsible for the aerobic degradation of phenol [9]. Except for the contig 55 (gene 3), that was classified as Cloroflexi, the annotation results revealed that all sequences were affiliated to the Proteobacteria phylum, being the Betaproteobacteria class the most abundant one. Literature data have shown that some representatives of this class, such as *Acidovorax* spp. [26], *Comamonas testosteroni* [27] and *Azoarcus* spp. [28], are species able to degrade pollutant compounds, such as toluene and phenol.

**Table 1:** Annotation of protein sequences related to phenol degradation genes from metagenomic pyrosequencing data.

Name	Length (aa)	Superfamily	Most Similar Protein	Host Organism	AA identity(%)	Bacterial Division
Contig6(gene2)	421	MmoB/DmpM	Monooxygenase subunit P2	*Pseudomonas putida* GB-1	82/89(92%)	γ-proteobacteria
Contig279(gene2)	515	Ferritin Like	Phenol hydroxylase subunit P3	*Azoarcus* sp. BH72	399/516(77%)	β-proteobacteria
Contig281(gene2)	515	Ferritin Like	Phenol hydroxylase subunit P3	*Azoarcus* sp. BH72	399/516(77%)	β-proteobacteria
Contig20(gene17)	519	Ferritin Like	Phenol hydroxylase subunit P3	*Comamonas testosteroni* CNB-2	426/512(83%)	β-proteobacteria
Contig6(gene1)	576	Ferritin Like	Phenol hydroxylase subunit P3	*Marinobacter algicola* DG893	320/376(85%)	γ-proteobacteria
Contig74(gene1)	501	Ferritin Like	Phenol hydroxylase subunit P3	*Acinetobacter radioresistens* SK82	432/501(86%)	γ-proteobacteria
Contig72(gene1)	576	P-loop NTPase	Positive regulator Phenol hydroxylase	*Azoarcus* sp. BH72	120/218(55%)	β-proteobacteria
Contig571(gene1)	226	P-loop NTPase	Positive regulator Phenol hydroxylase	*Azoarcus* sp. BH72	125/220(57%)	β-proteobacteria
Contig572(gene6)	569	P-loop NTPase	Positive regulator Phenol hydroxylase	*Azoarcus* sp. BH72	198/346(57%)	β-proteobacteria
Contig597(gene6)	589	P-loop NTPase	Positive regulator Phenol hydroxylase	*Azoarcus* sp. BH72	317/557(57%)	β-proteobacteria
Contig20(gene19)	581	P-loop NTPase	Positive regulator Phenol hydroxylase	*Azoarcus* sp. BH72	308/552(56%)	β-proteobacteria
Contig74(gene3)	594	P-loop NTPase	Positive regulator Phenol hydroxylase	*Azoarcus* sp. BH72	273/554(49%)	β-proteobacteria
Contig458(gene1)	452	P-loop NTPase	Positive regulator Phenol hydroxylase	Gamma proteobacterium IMCC3088	220/452(49%)	γ-proteobacteria
Contig36(gene1)	276	Ferritin Like	Phenol hydroxylase subunit P1	*Comamonas testosteroni* CNB-2	204/276(74%)	β-proteobacteria
Contig530(gene1)	310	Ferritin Like	Phenol hydroxylase subunit P1	*Comamonas testosteroni* CNB-2	235/310(76%)	β-proteobacteria
Contig74(gene2)	335	Ferritin Like	Phenol hydroxylase subunit P1	*Acinetobacter* sp. RUH2624	200/329(61%)	γ-proteobacteria
Contig20(gene18)	230	Ferritin Like	Phenol hydroxylase subunit P1	*Comamonas testosteroni* S44	234/328(71%)	β-proteobacteria
Contig20(gene16)	350	FNR Like	Oxiredutase FAD-binding P5	*Burkholderia multivorans* CGD1	255/350(73%)	β-proteobacteria
Contig87(gene2)	295	FNR Like	Oxiredutase FAD-binding P5	*Pseudomonas putida* GB-1	317/353(90%)	γ-proteobacteria
Contig496(gene1)	297	FNR Like	Oxiredutase FAD-binding P5	*Acinetobacter* sp. DR-1	212/297(71%)	γ-proteobacteria
Contig79(gene2)	308	Glo-EDI-BRP Like	Catechol 2,3-dioxygenase	*Acidovorax* sp. JS42	298/308(97%)	β-proteobacteria
Contig80(gene2)	308	Glo-EDI-BRP Like	Catechol 2,3-dioxygenase	*Alicycliphilus denitrificans* BC	288/308(94%)	β-proteobacteria
Contig20(gene15)	314	Glo-EDI-BRP Like	Catechol 2,3-dioxygenase	*Acidovorax* sp. JS42	269/314(86%)	β-proteobacteria
Contig55(gene3)	328	Glo-EDI-BRP Like	Catechol 2,3-dioxygenase	*Thermomicrobium roseum* DSM5159	122/308(40%)	Cloroflexi
Contig579(gene4)	308	Glo-EDI-BRP Like	Catechol 2,3-dioxygenase	*Alicycliphilus denitrificans* BC	273/308(89%)	β-proteobacteria
Contig581(gene4)	308	Glo-EDI-BRP Like	Catechol 2,3-dioxygenase	*Alicycliphilus denitrificans* BC	276/308(90%)	β-proteobacteria

**Figure 4:**
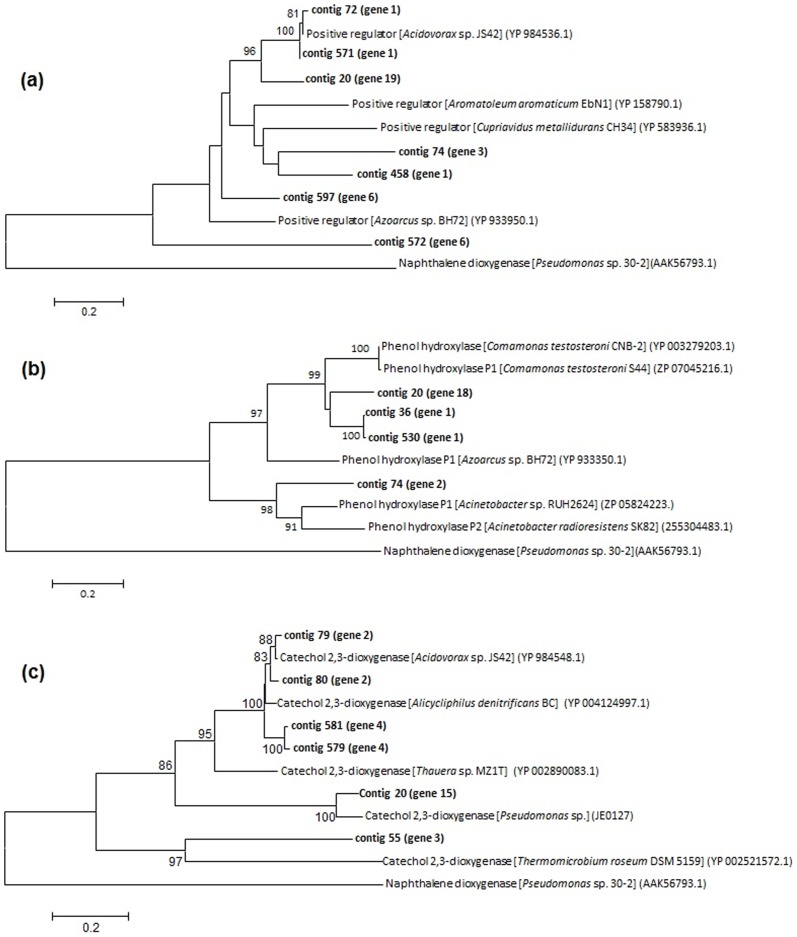
Neighbour-joining phylogenetic tree based on amino acid sequences of subunits of phenol hydroxylase and catechol 2,3- dioxygenase enzymes from metagenomic pyrosequencing data. (**a**) Phenol hydroxylase positive regulator, (**b**) Phenol hydroxylase sub. 1 and (**c**) Catechol 2,3-dioxygenase. All reference protein sequences used were obtained from *SwissProt protein* and *Refseq*_*protein*. The bootstrap values greater than 70% are listed.

Phylogenetic analyses using as reference *Swiss-Prot protein* and Reference protein (Refseq_prot) databases were done with contigs similar to catechol 2,3-dioxygenase and to some subunits of mPHs enzymes. The comparison of the amino acids sequences from these proteins with those previously deposited in the Genbank database revealed that some metagenomic sequences from the selected clones were clearly grouped separately from known sequences, including the phenol hydroxylase positive regulator, phenol hydroxylase subunit 1, oxireductase FAD-binding and catechol 2,3-dioxygenase (Figure 4, a to c). In the phylogenetic analyses with the positive regulator protein, the contigs 20 (gene 19), 74 (gene 3), 458 (gene 1), 597 (gene 6) and 572 (gene 6) formed a separate cluster. The formation of distinct clusters, distantly related to known sequences, was also verified for the phenol hydroxylase subunit 1, represented by the contigs 530 (gene 1), 36 (gene 1), 74 (gene 2). A closer look into the data showed that these sequences were affiliated to the Proteobacteria phylum. Contigs 20 (gene 16) and 496 (gene 1), related to oxidoreductase FAD-binding subunit, were clearly distantly related to known sequences, the phylogenetic reconstruction suggests that these sequences belongs to the Gamma- and Betaprotebacteria classes, respectivelly. Some contigs affiliated to catechol 2,3-dioxygenase formed distinct clusters as well, such as the contigs 80 (gene 2), 581 (gene 4) and 579 (gene 4), and the phylogenetic analysis strongly suggests that they belong to the Betaproteobacteria class (Figure 4c). Phylogenetic reconstruction performed with all the phenol degrading genes found in the metagenomic data suggest that the contigs showing low relatedness with known sequences potentially represent new sequences not previously deposited in databases.

**Figure 5:**
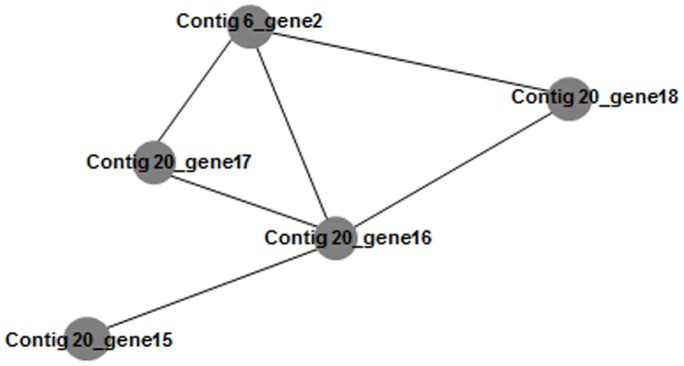
Network of predicted functional associations between proteins from contig 20 of metagenomic pyrosequencing data using STRING database. The circles represent phenol hydroxylase subunits and catechol 2,3- dioxygenase enzyme and the links between circles represent a putative interaction of these enzymes.

Some of the contigs, such as 20, 74, 579 and 581, were large enough to contain partial pathways. These contigs contained genes that encode phenol hydroxylase subunits and catechol 2,3- dioxygenase, enzymes required in the *meta*-pathway for degradation of phenol in aerobic bacteria, in addition to other aromatic compounds (e.g. toluene, xylene, cresol). The contig 20 was the largest one among them, containing five genes from the *meta*-pathway which encode for the C23O (gene 15), phenol hydroxylase oxidoreductase FAD-binding (gene 16), subunit 3 (gene 17), subunit 1 (gene 18) and positive regulator (gene 19). An in silico analysis was performed using the STRING database [24] to verify the interaction between the genes found in the contig 20, since they may be derived from a wide variety of microorganisms (Figure 5). Four of five genes (genes 15, 16, 17 and 18) predicted in contig 20 showed protein-protein interaction, and the gene 16 occupied the centre of the network, what might be explained by its role as a binding domain of the phenol hydroxylase enzyme. One gene (contig 20- gene19) was not included in the network analysis due to its low coverage (Table 1). The gene 2 of contig 6 was included in the network because it encodes the phenol hydroxylase subunit 2, which is not contained in contig 20. Although these genes originated from a metagenomic dataset, which implies that they may have come from different organisms, the network showed a clear interaction between phenol hydroxylase subunits and catechol 2,3- hydroxylase genes.

The contig 74 contained the genes that encode for the phenol hydroxylase positive regulator (gene 3), subunit 2 (gene 2) and subunit 3 (gene 1). Considering that the contigs were represented by strand -1, the genes codifying for the phenol hydroxylase positive regulator and subunits are upstream to the gene codifying for the C23O enzyme. This gene organization is corroborated by literature data that described the *meta*-pathway for *Pseudomonas* and *Acinetobacter* with similar organization [29], [30]. The 579 and 581 contigs were represented by genes encoding for the phenol hydroxylase subunit 3 (gene 2) and the catechol 2,3- dioxygenase (gene 4). Interestingly, the putative gene 3, between 2 and 4 genes, did not present similarity to any putative conserved domain available at the databases.

Except for the contig 55 (gene 3), that showed phylogenetic relationship to the Cloroflexi phylum, all contigs analyzed similar to catechol 2,3- dioxygenase and to some subunits of mPHs enzymes were affiliated to species belonging to the Proteobacteria phylum (Beta- or Gammaproteobacteria classes) (Table 1). These species have been reported to posses the metabolic pathways for a variety of different organic carbon sources. According to Mrozik *et al.* [31], several bacteria have been reported to harbor the metabolic pathways for the degradation of phenol, however the most effective bacteria are represented by strains from the genera *Burkholderia*, *Pseudomonas* and *Acinetobacter*. Bacteria of the genus *Acinetobacter*, such as *A. radioresistens* and *A. calcoaceticus* PHEA-2, use phenol or benzoate as sole carbon and energy source [32]–[34], being attractive candidates for the degradation of several pollutant compounds in bioremediation processes. El-Sayed *et al.* [35] showed that isolates of *Burkholderia cepacia* PW3 and *P. aeruginosa* AT2 could grow aerobically on phenol as sole carbon source, even at 3 g L^−1^ and both used the *meta*-cleavage activity of catechol 2,3- dioxygenase. The denitrifying bacteria group was also observed in the present study, exemplified by the *Azoarcus*/*Thauera* group use the aromatic compounds as *m*-xylene, phenol, toluene, ethylbenzene and others as carbon sources in anaerobic conditions [36]. Some strains of the genus *Alicycliphilus*, such as *A. denitrificans* BC and *A. denitrificans* K601, have been shown to degrade cyclic hydrocarbons [37]. Another denitrifying bacterium found in this work was *Aromatoleum aromaticum* EbN1, which is able to use a wide range of aromatic and nonaromatic compounds besides toluene, phenol, acetone and alcohols under anoxic and oxic conditions [38]. Some metal-resistant strains such as *Comamonas testosteroni* S44 and *Cupriavidus metallidurans* CH34 were also observed, the first bacterium shows high Zn(2+) resistance level and the last one is highly resistant to Zn(2+), Cd(2+) and Co(2+) [39]–[40]. Members of the genus *Comamonas* frequently occur in diverse habitats, including activated sludge, marshes, marine habitats, and plant and animal tissues [41]–[42]. Some species, such as *Comamonas testosteroni*, can also mineralize complex and xenobiotic compounds, such as phenol [43], testosterone [44], and 4-chloronitrobenzene (CNB) [45].

The metabolic and phylogenetic diversity observed in the petroleum wastewater microbial assemblages suggests that there is significant redundancy for phenol degradation within this environment. The enzymatic diversity identified in this study also revealed novel clades of enzyme classes that may be environmentally important in terms of phenol and aromatic compound degradation in wastewater treatment systems.

### Taxonomic classification, annotation and %GC content analysis of the fosmid insert

**Figure 6:**
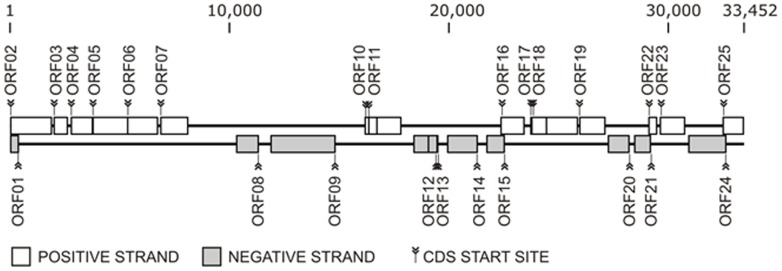
Schematic map of the gene cluster found in the fosmid insert. The squares indicate the predicted ORFs in positive (white) and negative (gray) strands, and marks indicate the coding sequence (CDS) start site. The annotation of ORFs is detailed in Table 2.

The assembly of the fosmid insert sequence was done using CLC Main Workbench software, under the most stringent parameters, and produced one single contig of 33.452 kb and 70.7% of GC content. This contig contained about 25 Open Reading Frames (ORFs), 15 positive- and 10 negative-stranded, which presented significant similarities to known proteins (Figure 6; Table 2).

**Table 2:** Predicted ORFs in the fosmid insert sequence.

ORF	Protein (GenBank Acession)	Host Organism	Bacterial Division	E-value
01	Tetratricopeptide TPR_2 repeat protein (ZP_11553451)	*Herbaspirillum frisingense* GSF30	β-proteobacteria	5,07E-07
02	Cysteinyl-tRNA synthetase (YP_316194)	*Dechlorosoma suillum* OS	β-proteobacteria	0,00E+00
03	HhH-GPD family protein (ZP_08930421)	*Thioalkalivibrio thiocyanoxidans* ARh 4	γ-proteobacteria	5,00E-63
04	Acetyl-coenzyme A carboxylase carboxyl transferase subunit alpha (ZP_18894696)	*Massilia timonae* CCUG 45783	β-proteobacteria	1,79E-170
05	tRNA(Ile)-lysidine synthetase (ZP_16730569)	*Pseudomonas syringae* pv. *aceris* str. M302273	γ-proteobacteria	5,23E-38
06	Aspartate kinase (ZP_11936099)	*Burkholderia* sp. TJI49	β-proteobacteria	0,00E+00
07	Leucine-binding protein (LBP) precursor (BAL26743)	*Azoarcus* sp. KH32C	β-proteobacteria	6,85E-95
08	Peptidase U35 (YP_465053)	*Anaeromyxobacter dehalogenans* 2CP-C	δ-proteobacteria	2,45E-06
09	DNA primase (ZP_05589090)	*Burkholderia thailandensis* E264	β-proteobacteria	3,00E-133
10	Transposase (YP_001632344)	*Bordetella petrii* DSM 12804	β-proteobacteria	5,00E-168
11	Transposition helper protein (YP_001792026)	*Leptothrix cholodnii* SP-6	β-proteobacteria	2,00E-52
12	Transposase (YP_001789811)	*Leptothrix cholodnii* SP-6	β-proteobacteria	4,00E-101
13	Transposase (YP_001789812)	*Leptothrix cholodnii* SP-6	β-proteobacteria	3,00E-57
14	Prophage CPS-53 integrase (ZP_18353285)	*Klebsiella pneumoniae* subsp. *pneumoniae* KpQ3	γ-proteobacteria	5,00E-67
15	PEP-CTERM putative exosortase interaction domain protein (AEI30474)	uncultured microorganism	-	1,85E-18
16	Dienelactone hydrolase-like enzyme (ZP_10697015)	*Pseudomonas* sp. GM21	γ-proteobacteria	7,00E-140
17	Acyl-CoA dehydrogenase (ZP_00955805)	*Sulfitobacter* sp. EE-36	α-proteobacteria	0,00E+00
18	TetR family transcriptional regulator (ZP_09975833)	*Mycobacterium phlei* RIVM601174	Actinobacteria	1,00E-14
19	ABC-type branched-chain amino acid transport system (ZP_11430428)	*Bradyrhizobium* sp. YR681	α-proteobacteria	3,00E-143
20	S-adenosylmethionine uptake transporter (YP_005188916)	*Sinorhizobium fredii* HH103	α-proteobacteria	8,00E-47
21	SOS-response transcriptional repressor (ZP_08330306)	*Gamma proteobacterium* IMCC1989	γ-proteobacteria	6,00E-28
22	Hypothetical protein (ZP_16011525)	*Neisseria meningitidis* NM3081	β-proteobacteria	2,54E-06
23	Short-chain dehydrogenase/reductase SDR (BAL26454)	*Azoarcus* sp. KH32C	β-proteobacteria	2,00E-67
24	Amidohydrolase 3 (YP_001411856)	*Parvibaculum lavamentivorans* DS-1	α-proteobacteria	0,00E+00
25	Propionyl-CoA carboxylase (ZP_16187627)	*Cupriavidus necator* HPC(L)	β-proteobacteria	3,00E-54

Sequence annotation was based on Blastx searches.

Twenty-two ORFs were annotated with significant similarities to known proteins of Proteobacteria, ORF18 was similar to a protein of Actinobacteria, ORF15 to a protein of an unknown organism, and ORF22 was similar to a hypothetical protein with unknown function (Figure 6; Table 2). Among the known proteins annotated, ORFs encoding the SOS-response transcriptional repressor (ORF21) and HhH-GPD family protein related to DNA repair (ORF3) were identified. Regulators of bacterial drug transporter were found in this fosmid insert, such as TetR families (ORF18) that regulate tetracycline efflux genes [46]. Genes related to mobile regions were also identified, such as three transposases (ORFs 10, 12, and 13), one transposition helper protein (ORF11) and one prophage CPS-53 integrase (ORF14).

Although the fosmid insert did not contain any complete known aromatic compound pathway, the dienelactone hydrolase-like enzyme (*clc*D), coded by ORF16, attracted attention since it belongs to the central catechol *ortho*-cleavage pathway. This enzyme plays a crucial role in the degradation of catechol and 3-chlorocatechol to tricarboxylic acid (TCA)-cycle intermediates [47]. The presence of the *clc*D gene in the fosmid insert could explain the ability of the host cell, *Escherichia coli* EPI300, to grow in the presence of phenol as sole carbon and energy source. Gaillard and collaborates [48] studied the *clc* element, a 103 kb genomic island originating in Pseudomonas sp. strain B13, showed that *Pseudomonas* species carrying the *clc* element acquired the capacity to grow on 3-chlorobenzoate and 2-aminophenol as sole carbon and energy substrates.

Additionally, the comparison of the whole fosmid sequence (33 kb) against bacterial genomes deposited in GenBank showed that only 10% of the fosmid sequence revealed identity with known proteobacteria sequences, suggesting that this genomic region may belong to a yet unidentified Proteobacteria (Table S1).

In summary, the screening for metagenomic clones with the ability for phenol degradation based on sequence and function revealed 26 and 413 positive clones, respectively. The sequencing of the positive fosmids showed the possibility of finding new genes and organisms responsible for phenol and aromatic compound degradation in refinery wastewater treatment system, highlighting the potential of such environment as a reservoir of enzymes for future application in biotechnological processes.

## Supporting Information

Table S1 **Top hits selected in Blastn searches, comparing the fosmid sequence with bacterial genomes deposited in GenBank.**
(DOC)
