# Supplementary material for: Identification of Genes and Pathways Related to Phenol Degradation in Metagenomic Libraries from Petroleum Refinery Wastewater
Source: PLoS One. 2013 Apr 18;8(4):e61811. doi: 10.1371/journal.pone.0061811 (PMC3630121; doi:10.1371/journal.pone.0061811)
Supplement: Table S1 — Top hits selected in Blastn searches, comparing the fosmid sequence with bacterial genomes deposited in GenBank. (DOC) [file pone.0061811.s001.doc]

**Supplemental Files**

**Table S1.** Top hits selected in Blastn searches, comparing the fosmid sequence with bacterial genomes deposited in the GenBank database.

| **GenBank Accession** | **Organism** | **Bacterial Division** | **Query coverage** | **Identity** | **E-value** |
| --- | --- | --- | --- | --- | --- |
| CP000555 | *Methylibium petroleiphilum* PM1 | β-proteobacteria | 11% | 97% | 0,00E+00 |
| CP002599 | *Burkholderia gladioli* BSR3 | β-proteobacteria | 10% | 95% | 0,00E+00 |
| FR854089 | *Ralstonia syzygii* R24 | β-proteobacteria | 11% | 95% | 0,00E+00 |
| FP885906 | *Ralstonia solanacearum* str. PSI07 | β-proteobacteria | 10% | 95% | 0,00E+00 |
| FP885897 | *Ralstonia solanacearum* CFBP2957 | β-proteobacteria | 11% | 96% | 0,00E+00 |
| CU914168 | *Ralstonia solanacearum* strain IPO1609 | β-proteobacteria | 11% | 96% | 0,00E+00 |
| CP002819 | *Ralstonia solanacearum* Po82 | β-proteobacteria | 11% | 96% | 0,00E+00 |
| CP001025 | *Burkholderia ambifaria* MC40-6 | β-proteobacteria | 11% | 97% | 0,00E+00 |
| CP003781 | *Burkholderia pseudomallei* BPC006 | β-proteobacteria | 11% | 97% | 0,00E+00 |
| CP001157 | *Azotobacter vinelandii* DJ | γ-proteobacteria | 9% | 95% | 0,00E+00 |
| CP000927 | *Caulobacter* sp. K31 | α-proteobacteria | 8% | 96% | 0,00E+00 |
| CP002418 | *Rhodopseudomonas palustris* DX-1 | α-proteobacteria | 11% | 100% | 0,00E+00 |
